# Quantitative assessment of lung mechanical properties in ARDS using X-ray computed tomography

**DOI:** 10.1186/s40635-026-00946-w

**Published:** 2026-07-23

**Authors:** Jian Gao, Roberta Garberi, Emmanuel A. Akor, Gaetano Perchiazzi, David W. Kaczka

**Affiliations:** 1 Carver College of Medicine, University of Iowa, Iowa City, IA USA; 2 Department of Anesthesia, University of Iowa, Iowa City, IA USA; 3 School of Medicine and Surgery, University of Milano-Bicocca, Monza, Italy; 4 Roy J. Carver Department of Biomedical Engineering, University of Iowa, Iowa City, IA USA; 5 The Hedenstierna Laboratory, Department of Surgical Sciences -- Anaesthesia and Intensive Care Medicine, Uppsala University, Uppsala, Sweden; 6 Department of Anesthesia, Operation and Intensive Care, Uppsala University Hospital, Uppsala, Sweden; 7 Department of Radiology, University of Iowa, Iowa City, IA USA; 8 Department of Anesthesia, University of Iowa Hospital and Clinics, 200 Hawkins Drive JCP 8483, Iowa City, IA 52242 USA

**Keywords:** Acute respiratory distress syndrome, Lung mechanics, X-ray computed tomography, Ventilator-induced lung injury, Deformable image registration

## Abstract

Acute Respiratory Distress Syndrome (ARDS) is marked by spatial heterogeneity in lung structure and mechanical behavior, limiting the ability of global physiologic measurements to guide ventilatory management and prevent ventilator-induced lung injury (VILI). X-ray computed tomography (CT) has emerged as a central imaging modality for characterizing regional differences in lung aeration, deformation, and mechanical properties that underlie such heterogeneity. When combined with quantitative image processing, deformable image registration, and computational modeling, CT enables regional assessment of lung strain, recruitment, and computationally inferred estimates of regional mechanical stress in injured lungs. This review summarizes CT-based methods for the quantitative evaluation of lung mechanical properties in ARDS, including image acquisition, segmentation, aeration analysis, deformable registration, and biomechanical modeling. Experimental and translational evidence is discussed to illustrate how CT has advanced the mechanistic understanding of regional ventilation distribution and deformation patterns that contribute to VILI. Methodological limitations and translational challenges are also discussed. Emerging directions, such as portable, low-dose, and photon counting CT are considered in the context of critically ill patients and their potential use for bedside assessment of lung mechanics.

## Take-home Message

In ARDS, lung structure and mechanics are highly heterogeneous, causing mechanical ventilation to generate uneven regional strains and stresses that are not captured by global respiratory mechanical measurements. Quantitative CT provides a regional view of lung aeration and deformation that helps explain the regional mechanisms underlying VILI.

140-character Tweet: CT imaging shows promise for quantifying regional lung mechanics in ARDS and potential risk factors for ventilator-induced lung injury.

## Introduction

Acute Respiratory Distress Syndrome (ARDS) remains a significant contributor to morbidity and mortality in critically ill patients [1]. As a highly lethal form of acute hypoxemic respiratory failure, ARDS is characterized by increased alveolar-capillary permeability, noncardiogenic pulmonary edema, severe hypoxemia, and bilateral pulmonary infiltrates [2, 3]. In addition, ARDS accounts for approximately 4 million ICU days annually in the U.S. alone, and is associated with mortalities of up to 40% [4]. Associated morbidities in survivors of ARDS can last for years, if not decades [5, 6]. The mainstay of early clinical management of ARDS often requires endotracheal intubation and supportive mechanical ventilation. However, a defining hallmark of ARDS is its marked structural and functional heterogeneity within the lung, resulting in significant differences in local tissue compliance, opening pressures, and time constants across different lung regions [7]. Despite decades of research, the ability to personalize ventilatory support and mitigate ventilator-induced lung injury (VILI) in ARDS patients remains limited [8, 9], owing largely to the heterogeneous and dynamic nature of lung injury. Such spatial heterogeneity challenges the implicit assumption that globally applied ventilatory settings exert uniform mechanical effects throughout the injured lung [10].

While recognition of VILI has driven many “lung-protective” strategies [2, 11–13], the pathophysiologic complexity of ARDS limits the effectiveness of standardized ventilatory settings. An important step toward understanding and addressing these clinical challenges is the quantitative assessment of lung mechanical properties, which directly reflect the physical state and functional capacity of the injured lung [14]. Ventilation and its associated mechanical forces are unevenly distributed in the lung with ARDS. Thus, regional ventilation and mechanical behavior must be considered alongside regional perfusion to support gas exchange, while simultaneously minimizing injurious mechanical loading [15]. Failure to account for this spatial heterogeneity may result in ongoing lung injury, even when low tidal volumes or limited driving pressures are applied [12, 16, 17].

Clinical assessment of respiratory mechanical function has traditionally relied on global inferences of lung function, usually derived from airway pressure and flow waveforms in ventilated patients [14]. Such inferences may include estimates of global respiratory resistance and compliance [18], driving pressures [12], mechanical power [16], as well as other waveform features indicating parenchymal overdistention and recruitment [19]. However, such global measurements fail to provide insight into the substantial regional heterogeneity that characterizes ARDS pathophysiology [7, 10].

Medical imaging now plays a critical role in guiding both diagnosis and management of ARDS [20, 21]. X-ray computed tomography (CT) has provided investigators with detailed assessments of structural and functional derangements over the clinical course of lung injury, and has emerged as a primary imaging modality for evaluating spatial variations in lung aeration and mechanics [22, 23]. X-ray CT provides high-resolution spatial volumetric data, which when combined with advanced image processing techniques, enables quantitative assessment of both regional and global ventilation, parenchymal deformation, and local lung tissue mechanics [24]. These image processing techniques expand the use of CT beyond simple anatomic or structural assessments, allowing multidimensional reconstructions of lung function at the regional level. CT imaging also permits the identification of regional differences in aeration, tissue texture, and parenchymal deformation, all of which are otherwise obscured by global measurements [25]. In contrast to other imaging modalities, CT yields the highest spatial resolution currently available for comprehensive assessment of regional lung structure and function [20, 22].

This review summarizes the use of CT imaging to characterize and quantify the mechanical properties of the lung during ARDS. While several recent reviews have summarized the role of imaging in ARDS diagnosis, phenotyping, and clinical management [20, 22, 26–28], the present paper focuses specifically on quantitative CT-based assessment of regional lung mechanics. We emphasize deformable image registration, regional strain and compliance analysis, texture-based characterization, and computational modeling approaches, all of which connect regional structure to mechanical function. By integrating such methodological and physiologic perspectives, this review highlights how CT can be used not only to characterize lung morphology, but also to infer regional mechanical processes implicated in ventilator-induced lung injury. We provide an overview of image processing approaches that have improved the ability of CT to characterize regional lung function and heterogeneity in ARDS. We also discuss current challenges and future directions for the use of CT in understanding ARDS pathophysiology and informing more precise, patient-specific strategies for mechanical ventilation.

## Regional lung mechanics

The distribution of ventilation (i.e., how gas is distributed during inflation and deflation) is determined by the lung’s regional mechanical properties [29–31]. Even healthy lungs exhibit regional variability in parenchymal mechanical time constants, due to the combination of the nonlinearity of alveolar pressure-volume relationships and exposure to varying intrapleural pressures [32, 33]. Such regional differences in lung mechanics become more pronounced in ARDS, because of the distribution of inflammation and its source [34], as well as the interplay between fluid accumulation and gravitational forces [35].

**Fig. 1:**
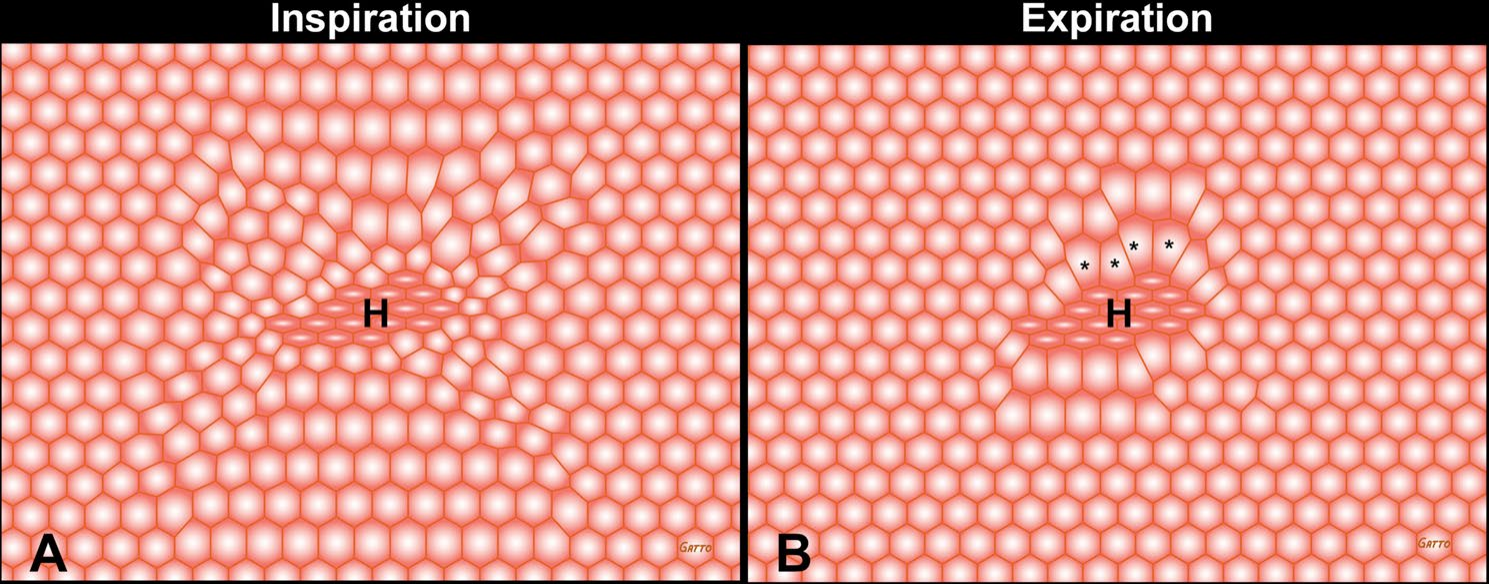
Alveolar interdependence provides a mechanistic basis for uneven local deformation in the heterogeneous injured lung. Schematic representation of interdependent alveoli at (A) inspiration and (B) expiration shows a central group of heterogeneously (H) collapsed alveoli that distorts neighboring aerated units because of shared walls. In the heterogeneous injured lung, this interaction can concentrate deformation within nearby open units, helping explain why regions of elevated strain or stress may coexist with collapsed or poorly ventilated lung units even when global parameters fall within ranges conventionally considered protective. Reproduced from Nieman et al. [39], with permission

Mead et al. introduced the concept of mechanical interdependence in the lung, in which individual alveoli do not inflate and deflate as independent units, but rather share stresses through common septa [36]. They based their analysis on the application of Hooke’s law to the three-dimensional structure of the lung, which relates force to spring elongation [37], thus reintroducing the classical concepts of stress and strain in the lung parenchyma [38]. Lung parenchymal strain refers to the elongation of lung tissue relative to its resting state at functional residual capacity (FRC). By contrast, lung stress describes the forces on the airway and alveolar walls, which is approximately equal to transpulmonary pressure in a mechanically homogeneous lung. However in the mechanically heterogeneous lung of ARDS, such interdependence may result in the amplification of local distending pressures, making them significantly higher than global transpulmonary pressure [36]. Figure 1 illustrates the conceptual basis for this local amplification of deformation through alveolar interdependence [39]. Evaluating potential parenchymal injury requires considering both stress and strain as key physical factors affecting lung structures [40, 41]. Such regional mechanical effects help explain why global ventilator variables do not fully capture local injury patterns and provide a more physiological rationale for CT-based regional assessment of lung mechanics.

### Quantitative assessment of lung mechanical properties during ARDS

#### Acquisition

The regional heterogeneity of ARDS motivates the use of imaging approaches to study regional lung structure and function [42, 43]. CT is an imaging modality that uses X-rays to produce a reference standard for assessing regional lung aeration and structural heterogeneity [20, 22]. Its sensitivity for detecting subtle or complex structural abnormalities surpasses that provided by plain chest radiography or bedside ultrasound [22]. Compared with other imaging modalities used in ARDS, CT offers a unique combination of whole-lung coverage and high spatial resolution, enabling quantitative assessment of regional lung structure at tissue spatial scales relevant to mechanical heterogeneity and VILI.

**Fig. 2:**
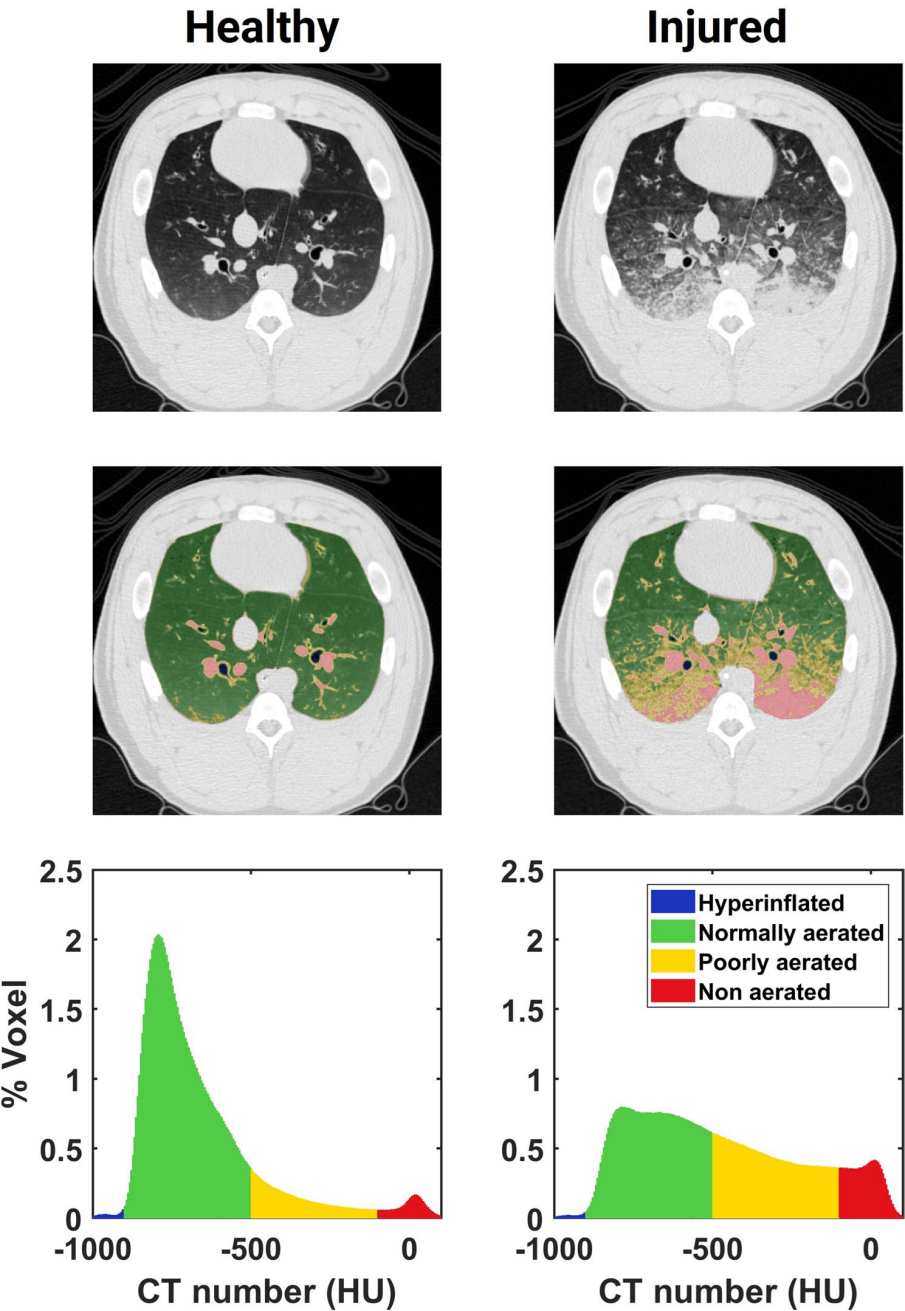
Regional lung aeration and corresponding CT density distributions in a representative pig, before and after an acute injury model similar to ARDS. Axial computed tomography (CT) slices are shown for two conditions (left: baseline; right: post-injury). The top row displays the original CT images. The middle row shows the corresponding voxel-wise classification of lung aeration overlaid on the CT images, based on Hounsfield unit (HU) thresholds: hyperinflated (− 1000 to − 901 HU, blue), normally aerated (− 900 to − 501 HU, green), poorly aerated (− 500 to − 101 HU, yellow), and non-aerated (− 100 to 100 HU, red). The bottom row presents the corresponding histograms of CT density distribution within the segmented lung regions, expressed as percentage of total lung voxels. Each histogram is partitioned according to the same aeration compartments, allowing direct quantitative comparison of the relative contribution of each class. Compared to baseline, the post-injury condition shows a rightward shift of the distribution, reflecting loss of normally aerated tissue and an increase in poorly and non-aerated compartments, consistent with increased lung density and reduced aeration

Modern CT scanners employ rotating X-ray sources and detector arrays to acquire volumetric datasets, with sub-second acquisition times and progressively reduced radiation exposures that are approaching those of chest radiographs. CT images for medical purposes are usually stored in the standardized format known as DICOM, which contains formatted image data and a detailed header containing the CT acquisition parameters [44]. Images are reconstructed into voxel-based maps of X-ray attenuation, expressed in Hounsfield units (HU), where air registers at − 1000 HU, water at 0 HU, and lung parenchyma spans intermediate values. Assuming each voxel is composed of a varying combination of these two components [42], it is possible to compute the percentage of gas in each voxel by applying a simple linear equation first introduced by Gattinoni [45]. Figure 2 shows an example of the regional heterogeneity in lung aeration in a pig, before and after the development of an acute injury similar to ARDS.^1^ To obtain the absolute quantity of gas, it is necessary to know the dimensions of each voxel, which in modern scanners are defined by the operator and reported in the DICOM header. The number of alveoli in each voxel depends on the dimension of the voxel, the species being imaged, and the degree of inflation of the alveoli when the image is acquired [46]. A voxel of about 1 mm³ corresponds to approximately 160 alveoli, enabling regional, but not alveolar-level, resolution [22].

**Fig. 3:**
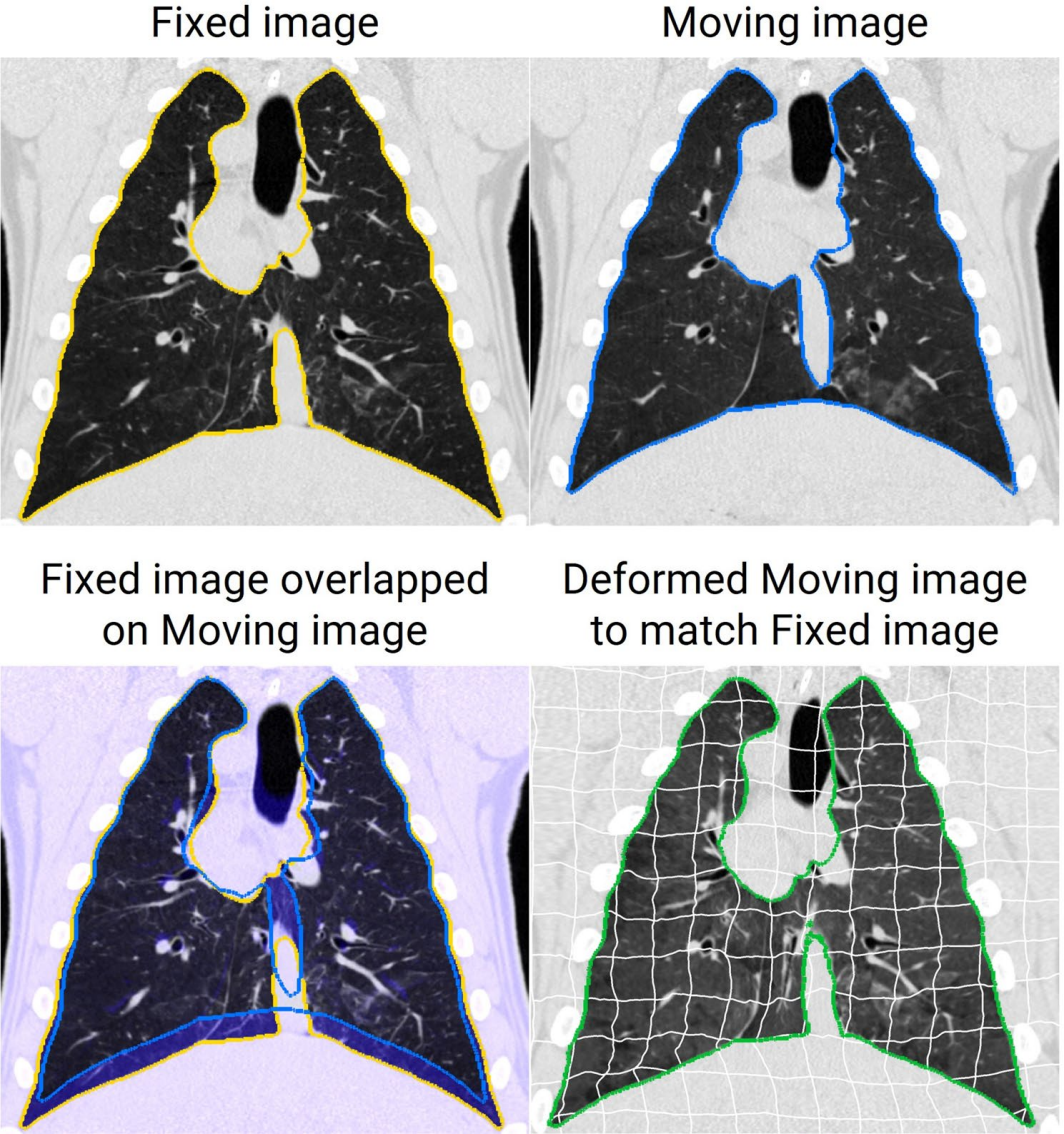
Illustration of deformable image registration. Representative coronal CT slice at two airway pressures is shown. From left to right: fixed image, moving image, overlay before registration, and warped image after registration. Lung masks are overlaid for each condition (fixed: yellow; moving: blue; warped: green). The pre-registration overlay demonstrates geometric mismatch between lung configurations at different pressures. Following deformable registration, the moving image is mapped onto the fixed image. The deformed grid visualizes the underlying displacement field, providing an intuitive representation of regional lung deformation

**Fig. 4:**
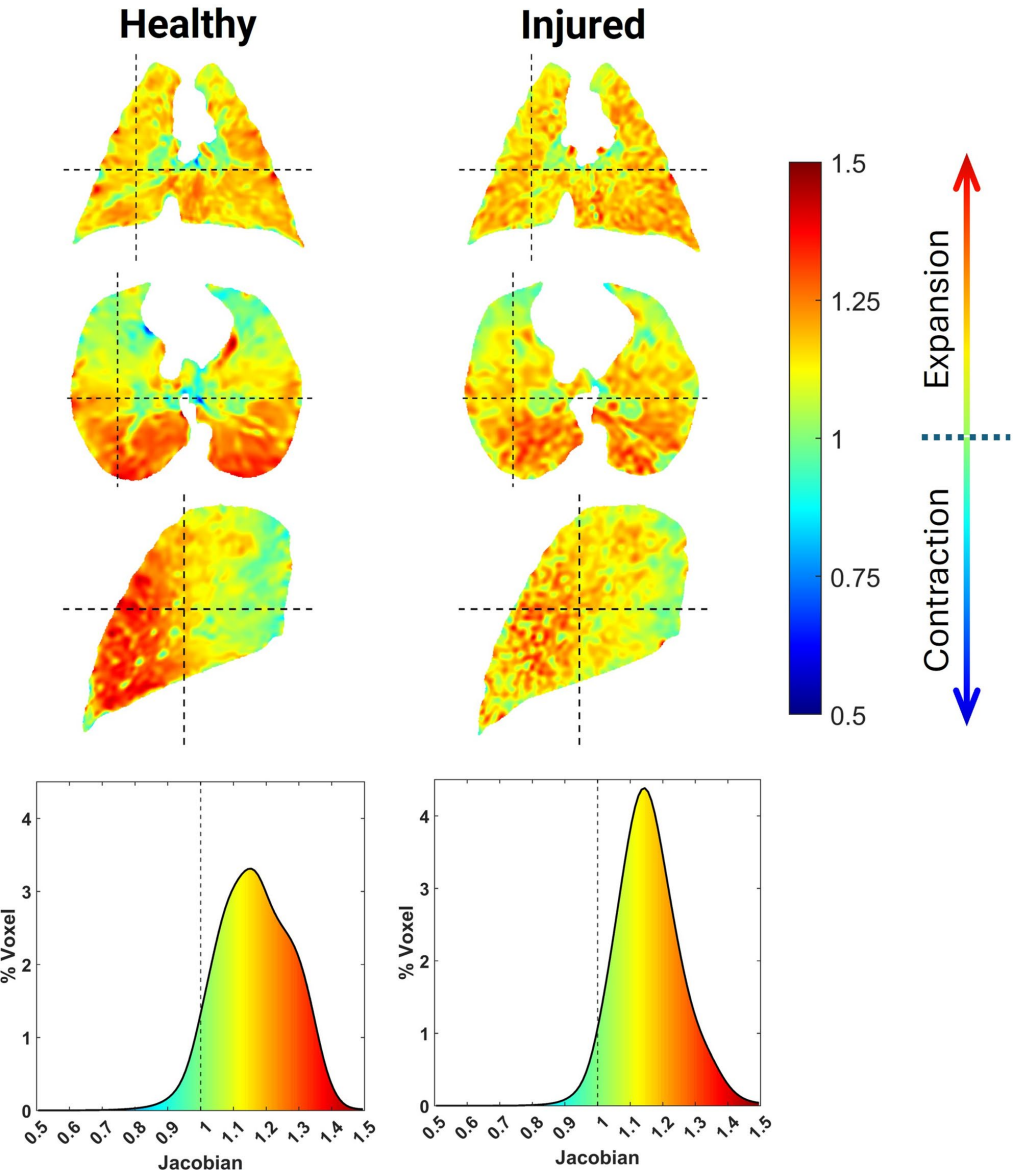
Regional lung expansion from 10 to 15 cmH_2_O assessed by the Jacobian determinant. Representative sagittal (top), coronal (middle), and axial (bottom) views of the lung are shown for two conditions (left: healthy, right: post-injury). Each panel displays the spatial distribution of the Jacobian determinant derived from deformable image registration, quantifying local volumetric expansion between airway pressures of 10 and 15 cmH_2_O. Values > 1 (yellow–red) indicate regional expansion, values ≈ 1 (green) indicate no volume change, and values < 1 (blue) indicate reduced expansion or local contraction. Dashed black lines indicate the positions of the corresponding orthogonal slices across views. The distributions of Jacobian values within the lung are shown in the lower panels for each condition, expressed as percentage of voxels

The spatial resolution of conventional clinical CT remains insufficient to directly resolve individual alveoli or alveolar septa. Experimental modalities such as micro-CT [47] and synchrotron phase-contrast imaging [48] can achieve substantially higher spatial resolution, and have provided unique insights into alveolar recruitment, micromechanical deformation, and tissue interdependence. Although currently limited to research applications, these techniques may link whole organ-scale CT measurements with alveolar-scale mechanisms of injury.

Although CT remains the gold standard for regional structural assessment in ARDS, other imaging modalities can also provide complementary information. Electrical impedance tomography (EIT) offers bedside, radiation-free monitoring of regional ventilation distribution with excellent temporal resolution, albeit at substantially lower spatial resolution. Magnetic resonance imaging (MRI) can provide functional information regarding ventilation, perfusion, and gas exchange without ionizing radiation, but remains technically challenging in critically ill patients. Positron emission tomography (PET) enables assessment of regional inflammation and metabolism but requires radiotracers and specialized facilities. Lung ultrasound (US) provides rapid bedside assessment of aeration and pleural abnormalities, but is often artifact-based and lacks comprehensive volumetric coverage. Despite their limitations, these complementary imaging modalities may link detailed structure-function characterization, and for EIT and US, continuous bedside monitoring.

#### Lung segmentation

Following initial image acquisition, a required step before characterizing regional quantitative metrics of the lung is lung segmentation. This is generally a straightforward procedure for healthy lungs, given the sharp difference in Hounsfield density between the parenchyma and the surrounding structures of the chest wall and mediastinum. Such sharp contrast allows the use of simple threshold-based approaches for segmentation [49], which may subsequently require minor manual corrections [50]. However, lungs with ARDS are characterized by areas of high Hounsfield density, due to various combinations of local atelectasis, edema, and inflammation, which often preclude such semi-automated approaches. In more recent years, the availability of deep learning techniques has allowed segmentation to be performed rapidly and reliably in injured lungs using convolutional neural networks [51–53].

#### Lung registration

After obtaining an image of the lung without surrounding anatomic structures, it is possible to begin additional quantitative regional analyses. In many cases, it is desirable to compare the segmented lung image to another image obtained at a different inflation pressure or gas volume, or when combining imaging modalities (i.e., PET-CT or MRI-CT), at another resolution, inflation state, or projection. Registration is the process by which lung images may be aligned across varying states of time, pressure, or volume [54, 55], determining a spatial transformation that maps points in one image (i.e., the “moving” image) to corresponding locations in another image (i.e., the “reference” image) (Fig. 3). Numerous approaches have been proposed to address this problem employing different combinations of registration algorithms to accommodate varying degrees of morphological disparity between the images under analysis. For lungs with ARDS, this mapping enables voxel-level tracking of tissue between inflation states or time points, allowing regional deformation and strain to be directly quantified [50, 56–59]. Deformable (i.e., non-rigid) registration is generally preferred for lung imaging, because it accounts for the complex and spatially heterogeneous deformation of the lung during inflation and deflation, unlike rigid registration, which is limited to translation and rotation.

With deformable registration, an individual volume element *n* (i.e., voxel) at one inflation pressure or time point is mapped to a new location at another inflation pressure or time point [25, 50, 60]. The output is the deformation field, that is, a vector field describing the local displacement required to bring the moving image into alignment with the reference image. From this deformation field, local expansion and compression can be estimated across the lung. A commonly used summary metric is the Jacobian determinant of the deformation field, |**J**_***n***_|, which represents the local volume ratio between two states and can be interpreted as a measure of regional volumetric strain [25, 50, 57, 59]. Values greater than 1 indicate local expansion, while values less than 1 indicate local compression. Values near 1 indicate little change in voxel volume. Figure 4 shows example CT-based anatomic maps of |**J**_***n***_| in porcine lungs.

When evaluated across the whole lung, Jacobian maps visualize regional patterns of expansion and compression and can highlight areas in which tidal deformation is concentrated within the remaining aerated lung. Conversely, regions with low deformation may correspond to poorly aerated or consolidated compartments that may undergo cyclic recruitment at interfaces with adjacent aerated tissue [58, 59]. Using commercially available software and applying the relation introduced by Pelosi et al. [35], it is also possible to compute superimposed pressures at the voxel level. This method relies on estimating the regional pleural pressure by assessing the position of the esophageal catheter relative to the vertical axis of the lung. Applying the same principle and calculating the map of the volume difference between two consecutive inflation states, it is possible to determine regional lung compliance on a voxel-by-voxel basis [57].

Validation of deformable registration remains an important consideration in quantitative CT imaging, particularly for severely injured lungs where large regional density changes and tissue collapse may complicate image correspondence. Registration accuracy is commonly assessed using manually identified anatomical landmarks, inverse-consistency testing, or comparison against reference datasets containing expert-defined correspondences [56, 60]. Such validation approaches help ensure that derived deformation fields provide physiologically meaningful characterization of regional lung mechanics.

Respiratory-gated dynamic CT has also been used to quantify regional deformation across the breathing cycle before and after lung injury in large animal models [61]. Deformable registration between successive respiratory phases allows calculation of |**J**_***n***_| at each voxel, quantifying how much each small lung region expands or contracts during the breath, thereby providing a measure of local strain. Following injury, strain distribution became more heterogeneous, with abnormal regional deformation patterns emerging despite similar global ventilator settings [59]. Jacobian-based registration metrics may also be tested in clinical cohorts, for which the endpoint is not solely short-term physiological metrics, but also their association with more meaningful outcomes [62]. For example, a recent study in COVID-ARDS evaluated whether CT registration-based regional functional parameters relate to survival [63].

Another approach to estimate regional volume change is based on changes in regional aeration using mass-balance principles, linking deformation to local gas and tissue fraction within each voxel [56, 64]. Whereas Jacobian-based metrics emphasize geometric deformation of tissue, aeration-based approaches emphasize changes in local gas content. Applying both approaches in parallel can cross-check regional strain estimates [64], which is especially important in ARDS, since significant heterogeneity in structure may complicate interpretation of regional function.

A final method is offered by translational processing models that tolerate high radiation doses, while scanning multiple lung images over small volume increments [25, 57]. This approach minimizes the effects of the unpredictable nonlinear motion of lung portions subjected to heterogeneous attraction forces. This small volume increment allows one to consider two consecutive images as differing by multiple local linear movements, and to solve the transformation by applying algorithms based on the so-called optimal triangulation of control points [65].

Despite recent advancements in image registration, several technical challenges remain. Registration accuracy may be affected by motion, large interval changes in lung volume, image quality, and partial volume effects, especially near interfaces between aerated and nonaerated tissue [56, 66]. Deformable registration can also be computationally demanding, especially for large four-dimensional CT datasets acquired across multiple respiratory phases [59]. At present, these constraints limit routine clinical implementation, restricting CT-based registration techniques primarily to basic and translational research.

#### Density analysis and dual energy CT

The most common analysis of lung images is based on the direct use of CT density expressed as HU. In this respect, lung parenchyma can be classified into four functional compartments (hyperaerated (< − 901 HU), normally aerated (–900 to − 501 HU), poorly aerated (–500 to − 101 HU), and non-aerated (> − 100 HU ) as originally proposed by Gattinoni [67]. These methods enable calculation of aerated and non-aerated lung volume, recruitability, and responses to ventilator settings. In this way, it was possible to reveal that lung injury is heterogeneous [67–69], often showing a ventral-dorsal gradient with increased density and consolidation in dependent regions and relatively better aeration in nondependent regions. As illustrated in Fig. 2, regional heterogeneity based on CT density is characterized by dependent loss of aeration and relative sparing of nondependent lung. These qualitative CT observations helped establish the concept of the “baby lung”, namely the reduced fraction of aerated lung tissue available for gas exchange [70].

**Fig. 5:**
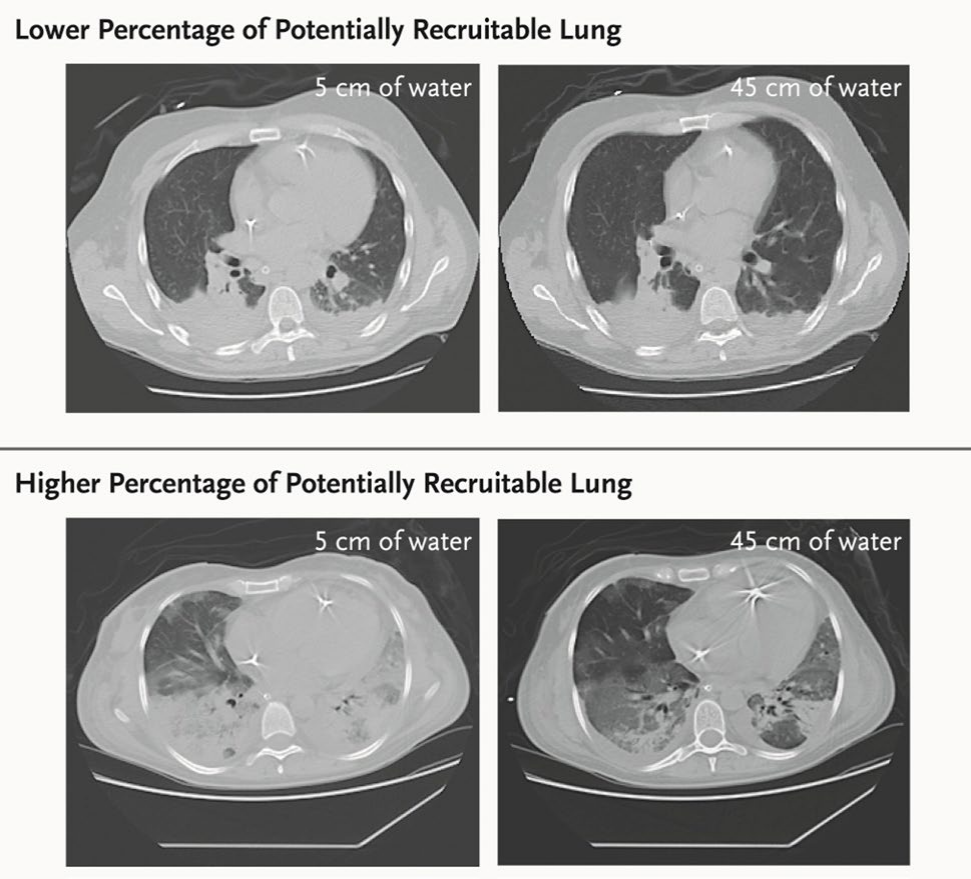
CT-based assessment of lung recruitability in human ARDS. Representative thoracic CT images obtained at low (5 cm H₂O) and high (45 cm H₂O) airway pressures demonstrate examples of high and low recruitability. Patients with highly recruitable lungs (bottom panels) show substantial reaeration of previously collapsed dependent regions with increasing airway pressure, whereas patients with low recruitability (top panels) exhibit relatively little change. These findings illustrate the marked interpatient heterogeneity of ARDS morphology and provide a structural basis for individualized ventilatory strategies. Adapted from Gattinoni et al. [71], with permission

While the baby lung represents the currently aerated functional lung volume, recruitable lung refers to previously nonaerated regions that may reopen with increased airway pressure or recruitment maneuvers. The distinction is clinically relevant, because mechanical ventilation is preferentially distributed to the baby lung. By contrast, “recruitability” reflects the potential for increasing functional lung size through ventilatory interventions. CT imaging has also demonstrated that the amount of potentially recruitable lung varies substantially among patients with ARDS. As shown in Fig. 5, some patients exhibit marked reaeration of dependent lung regions following increases in airway pressure, whereas others demonstrate little recruitment despite similar degrees of average CT density [71]. This variability highlights the heterogeneous mechanical behavior of lung with ARDS, and helps explain why uniform ventilatory strategies may produce different physiologic responses across patients. Such CT-based assessments of recruitability have also demonstrated that mechanical ventilation is preferentially distributed to this limited aerated lung volume, providing a framework for understanding why acceptable global ventilator settings may still produce injurious regional deformation in ARDS [13]. Moreover, the severity and morphological distribution of injury in lungs with ARDS may correspond to different patterns of mechanical heterogeneity, allowing ARDS to be classified into focal versus non-focal phenotypes, with potential implications for ventilator management [72].

Functional imaging extends CT beyond static morphology to characterize regional lung function and its temporal evolution. Such approaches include dynamic CT to evaluate intratidal recruitment and derecruitment [66], as well as dual-energy CT with inhaled or intravenous contrast to map ventilation and perfusion [73], thus providing insights into regional ventilation-to-perfusion matching. Hounsfield densities for soft tissues show little change with beam energy, but high atomic number materials such as iodine exhibit noticeable differences. Using different X-ray energies helps differentiate iodinated contrast media, which enhances more at low tube voltages. This allows dual-energy imaging to distinguish iodine from other substances in a single contrast phase, enabling targeted visualization of iodine in tissues such as the lungs [74]. In practice, a dual-energy scan during the administration of contrast media yields a simultaneous image of the distribution of the contrast agent (usually following the vascular tree), together with a ventilation map generated from |**J**_***n***_| derived from a virtual non-contrast image. In this way, it is possible to estimate the regional distribution of ventilation and perfusion from the directly measured aeration and blood content [73, 75].

#### Composite measurements and texture analysis

**Fig. 6:**
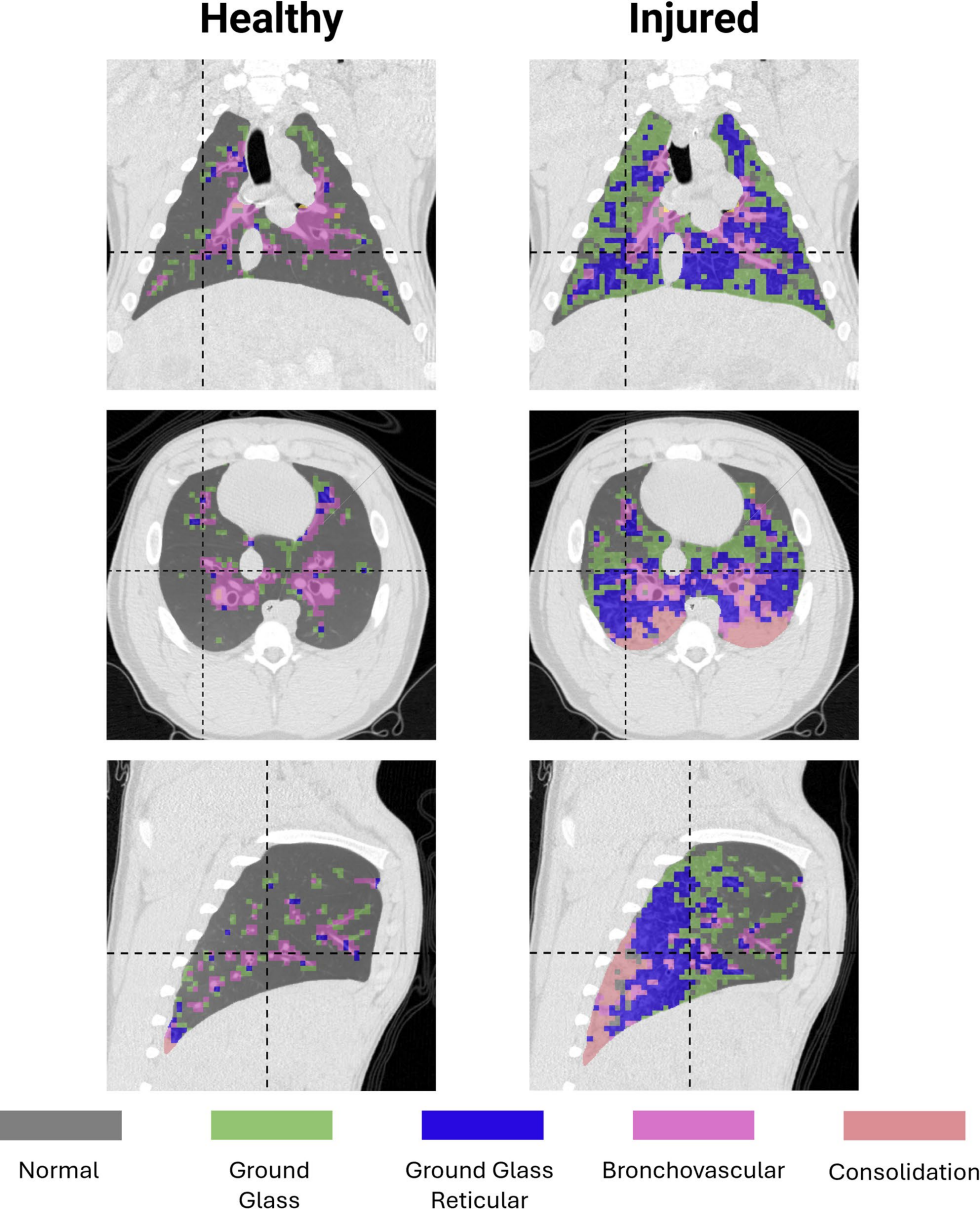
Regional distribution of lung texture classes derived from the Adaptive Multiple Feature Method (AMFM). Representative coronal (top), axial (middle), and sagittal (bottom) views are shown for two conditions (healthy, left; injured, right). The grayscale background represents the underlying CT image, while colored overlays indicate voxel-wise texture classes obtained using AMFM analysis. These classes capture regional variations in lung parenchymal structure and spatial heterogeneity beyond density-based measures. Display is restricted to voxels within the lung mask. Dashed black lines indicate the positions of the corresponding orthogonal planes across views

Combining CT imaging and global respiratory mechanics measurements (i.e., airway pressure and flow, esophageal pressure) allows for further discernment of structure-function relationships in the lung. Beyond density-based aeration analysis, CT texture analysis may provide additional characterization of regional structural heterogeneity in ARDS. Figure 6 shows how texture-based classification can quantify structural heterogeneity beyond density-based aeration analysis alone. Supervised texture classification, including the adaptive multiple feature method (AMFM), can categorize lung parenchyma according to local image features, allowing the identification of patterns such as normal, ground-glass, reticular, bronchovascular, or consolidated regions [76–78]. By incorporating regional image characteristics beyond voxel intensity alone, such approaches may detect abnormalities that are not fully captured by conventional Hounsfield-based aeration compartments [25].

Although the definition of morphological heterogeneity is intuitively understood by researchers, its numerical quantification is rarely addressed in the literature. A useful instrument for quantification of lung heterogeneity is to analyze anatomic maps of Hounsfield density, air volume, compliance, or other variables using a quadtree decomposition algorithm [79], a commonly used algorithm for image compression. With this technique, Perchiazzi et al. [57]. quantified the heterogeneity of the compliance field by computing the so-called mean homogeneous volume in healthy and injured lungs. Using a similar approach, Herrmann and co-workers quantified both the heterogeneity of regional parenchymal strain [59] and specific ventilation [80] during different modes of mechanical ventilation. More recently, Liggieri et al. described the computation of lung mechanical strain at the voxel level [81]. The variables used in the calculation were the same as those used to determine regional compliance, with the main difference being that the denominator is the volume of the corresponding voxel at FRC. The strain field calculated in this way is the normal strain (i.e., perpendicular to the faces of the ideal parallelepiped encompassing one voxel), and thus may not include shear strain (i.e., parallel to the surfaces of the voxel).

CT-based strain assessment of ARDS lungs at high spatial resolution raises important physiological concerns: individual voxels represent alveoli with widely varying inflation states and are not functionally connected to the airways. When voxels portray poorly inflated or non‑inflated alveoli at FRC, the denominator of the strain equations becomes very small, leading to artificially high strain values. This issue of non‑ventilated regions affecting voxel‑level strain has been recognized previously. Earlier studies attempted to correct this by adjusting the strain denominator using estimated recruited volumes, based either on assumed CT densities or average regional aeration [82, 83]. However, these approaches can introduce errors. Moreover, the evaluation of CT-computed strain must resolve a physiological dilemma regarding the relationship between gas content and the effective generation of elastic recoil. Micromechanical studies suggest that newly recruited alveoli may begin to fill with gas and are represented as “ventilated” voxels. Nonetheless, such alveoli may still be unfolding, and may not yet generate elastic recoil [84]. This observation also has important implications for the development of computational models of parenchymal deformation (see below). Such models may need to distinguish between gas-filled alveoli that actively contribute to tissue recoil, and newly recruited alveoli that remain mechanically disengaged during early inflation. Failure to account for this transitional state could lead to overestimation of regional compliance, and underestimation of local stress concentrations in ARDS.

#### Computational modeling

The integration of CT with computational modeling has further advanced our understanding of lung mechanics [31, 85–89]. CT provides regional structural and functional information that can serve as a foundation for forward and inverse models of lung mechanics [14]. Such modeling approaches can map mechanical properties of the whole lung with regional detail, thereby offering computational insights into recruitment, overdistension, parenchymal strain, and the distribution of regional ventilation and mechanical stress. Such quantitative modeling approaches are particularly pertinent in the context of protective ventilation strategies in ARDS [90]. By coupling regional CT data with computational models, it is now possible to assess how heterogeneous lung structure gives rise to nonuniform ventilation and stress patterns that may not be directly measurable at the bedside [7]. This improves our understanding of how heterogeneous lung mechanics contribute to VILI.

**Fig. 7:**
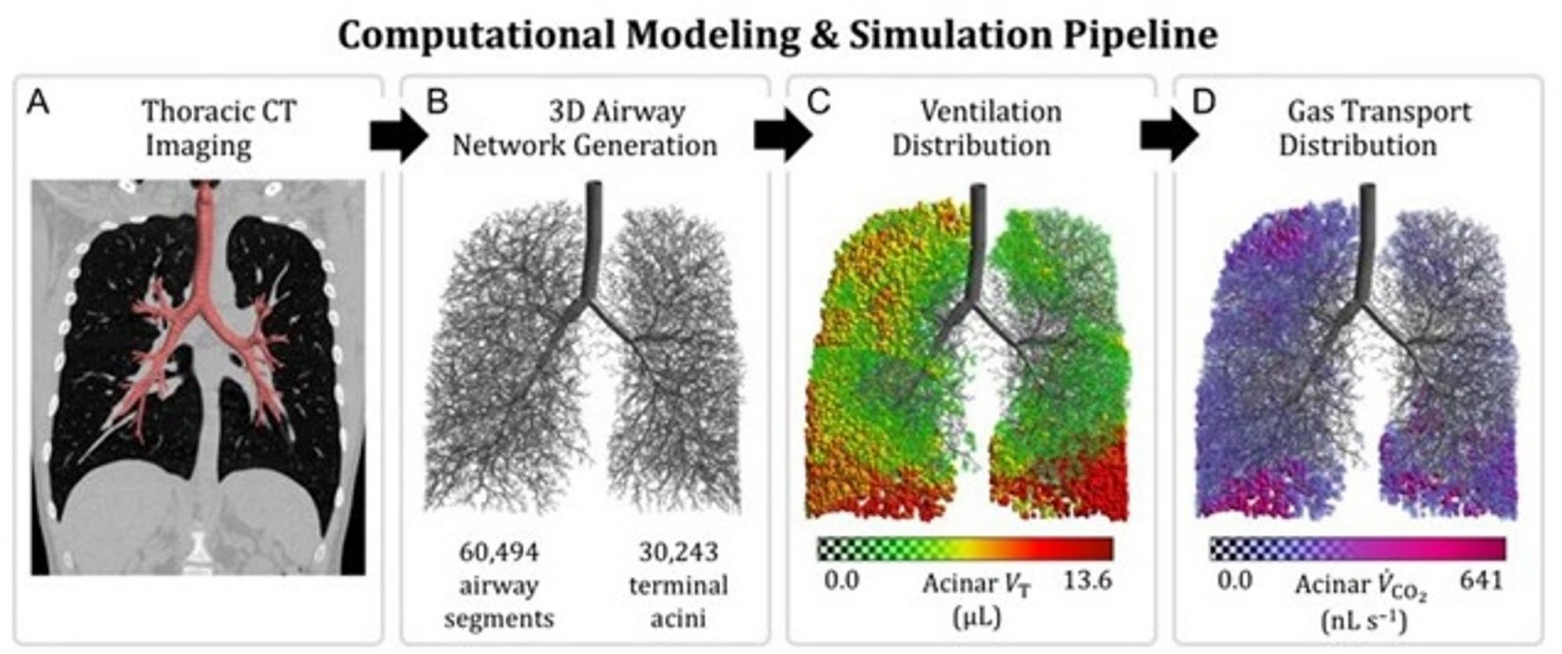
Computational processing pipeline for simulated ventilation distribution and gas transport in a computational model of primary blast injury. A thoracic CT image was segmented to obtain central airways (A), which were then used as initial conditions for a space-filling algorithm (B) to generate a three-dimensional network of smaller peripheral airways [94]. Models of mechanical impedance were applied to each airway segment and terminal acinus [31], to yield ventilation distribution in terms of acinar tidal volume, *V*_*T*_ (C). Gas transport models were then solved to find the necessary ventilation required to maintain eucapnic carbon dioxide elimination, $\:{\dot{V}\mathrm{C}\mathrm{O}}_{2}$ (D). Reproduced from Herrmann et al. [95], with permission

A typical CT-informed modeling workflow begins with segmentation of the lung and airways [51–53, 91], followed by the construction of a geometric mesh that represents the patient-specific anatomy [87, 89, 92]. Regional CT-derived information, including aeration, density, deformation, and recruitability, may then be used to parameterize local mechanical properties within the model [31]. Boundary conditions representing dynamic airway pressures, pleural pressures, or ventilator settings are subsequently applied, allowing forward simulations of ventilation distribution, regional strain, stress, and recruitment dynamics [90, 93, 94]. Inverse approaches may also be used to estimate regional mechanical parameters from measured physiologic data [50]. Figure 7 illustrates one potential simulation pipeline for a CT-informed computational model of regional ventilation distribution and gas exchange [95]. Together, these approaches transform static or dynamic CT information into mechanistic predictions of lung behavior under different ventilatory conditions.

Computational modeling is particularly valuable when key mechanical variables cannot be obtained directly from imaging alone, such as regional intraparenchymal stress, which is typically inferred through physiologic assumptions about pleural pressure, chest wall constraints, and force distribution throughout the lung, rather than direct measurement [17, 90]. CT-informed simulations can thus extend descriptive imaging to hypothesis testing, by evaluating how changes in ventilatory strategy redistribute regional deformation, recruitment, and inferred stress [96], which may ultimately support more individualized approaches to ventilator optimization [92]. Nonetheless, increasing the detail and accuracy of parameterized mechanical properties in computational lung models is also accompanied by increases in computational time and resources. Consequently, high-performance computing may be needed to enable clinical translation of these computational simulations.

## Challenges and future directions

Despite its strengths, CT-based assessments of regional lung properties have important limitations, including radiation exposure (~ 7 mSv for conventional thoracic CT), which constrains repeated imaging [97]. Although newer low-dose acquisition and reconstruction methods may substantially lower the delivered dose of ionizing radiation [22], concerns regarding cumulative exposure remain relevant, particularly in studies requiring serial imaging. In addition, there are risks associated with the use of contrast, including nephrotoxicity and hypersensitivity reactions. Patient transport to an imaging suite also poses a risk, with adverse events reported in up to 70% of cases during ICU-to-radiology transfers [98].

Photon-counting CT represents another promising technological development for quantitative lung imaging [99]. Unlike conventional energy-integrating detectors, photon-counting systems directly measure individual X-ray photon energies, enabling improved spatial resolution, reduced electronic noise, enhanced soft-tissue contrast, and intrinsic spectral imaging capabilities [100]. These features may improve quantification of regional lung density, perfusion, and tissue composition while simultaneously reducing radiation dose [101]. As clinical availability increases, photon-counting CT may further expand opportunities for functional and quantitative imaging in ARDS.

Substantial computational demands are required for quantitative and functional lung imaging analyses, although these continue to improve with advances in high-performance computing. Accurate image segmentation, deformable registration, and model-based analysis may be particularly challenging in severely injured lungs, where heterogeneous aeration, motion artifact, and changing lung geometry complicate interpretation. While CT imaging provides rich physiologic information on regional mechanics, evidence that CT-guided personalized ventilation improves clinical outcomes remains limited and has not demonstrated a mortality benefit over conventional low-tidal-volume approaches [20, 72]. However, several developments may help address these limitations. Continued reductions in radiation dose, improvements in acquisition speed, advances in automated segmentation and registration, and increasing integration of machine learning and radiomics could improve the feasibility and clinical utility of quantitative CT in ARDS [24]. More broadly, CT-informed phenotyping and patient-specific modeling could help identify subgroups of patients with distinct patterns of recruitability, regional strain, or mechanical vulnerability. Future work will determine whether such mechanistic insights can be translated into reproducible ventilatory strategies that improve clinical outcomes in ARDS.

## Conclusions

Quantification of lung mechanics using X-ray CT has matured from proof-of-concept studies to established tools for both experimental and translational research. Moreover, CT imaging has substantially advanced our understanding of regional lung heterogeneity in ARDS and the mechanical mechanisms underlying VILI. By linking density changes, deformation fields, and biomechanical models, CT enables mapping of regional ventilation, strain, and compliance with high spatial resolution. Ongoing advances in imaging hardware, computational algorithms, and analysis frameworks continue to strengthen the role of quantitative CT in mechanistic and translational ARDS research. The integration of CT-based mechanical phenotyping with serum biomarkers may provide complementary insight into early mechanisms of lung injury [41]. Portable cone-beam CT systems may enable bedside assessment of lung mechanics in intensive care units, reducing the risks associated with transporting critically ill patients [102]. However, current systems generally exhibit lower signal-to-noise ratios, increased scatter sensitivity, and less reliable Hounsfield unit calibration compared with conventional multidetector helical CT scanners. These limitations may affect quantitative assessments of regional aeration and deformation, although continued improvements in detector technology and image reconstruction algorithms are narrowing this gap.

CT imaging has been central to shaping our current understanding of ARDS and VILI, revealing the heterogeneous nature of lung injury and providing detailed physiologic insight. While its routine use in ventilator titration remains constrained by radiation exposure, patient transport, and limited evidence of outcome benefit, CT remains a central research tool and a valuable adjunct in complex clinical scenarios. Emerging technologies in portable imaging, photon-counting, and computational analysis hold promise for translating CT-derived insights into reproducible and more precise approaches to ventilatory support for critically ill patients.

## Data Availability

Data in this paper is available upon reasonable request to the corresponding author.

## Footnotes

1 The imaging data shown in Figs. 2, 3 and 4, and 6 are from a single porcine subject, which was part of an ongoing pre-clinical study (U.S. Department of Defense Award W81XWH-21-1-0507, University of Iowa IACUC Protocol 1022371). The subject was endotracheally intubated, under general anesthesia and neuromuscular blockade during imaging. Lung injury was induced with an infusion oleic acid into the right atrium (0.08 mL kg^− 1^).
